# Immunoregulatory soluble CTLA-4 modifies effector T-cell responses in systemic lupus erythematosus

**DOI:** 10.1186/s13075-016-1075-1

**Published:** 2016-08-04

**Authors:** Lekh N. Dahal, Neil Basu, Hazem Youssef, Rahul C. Khanolkar, Robert N. Barker, Lars P. Erwig, Frank J. Ward

**Affiliations:** 1 Section of Immunology and Infection, Division of Applied Medicine, Institute of Medical Sciences, University of Aberdeen, Foresterhill, Aberdeen, AB25 2ZD UK; 2 Department of Rheumatology, Aberdeen Royal Infirmary, Aberdeen, UK; 3 Renal Unit, Aberdeen Royal Infirmary, Aberdeen, UK; 4 Antibody and Vaccine Group, Cancer Sciences Unit, Faculty of Medicine, University of Southampton, Southampton General Hospital, Southampton, UK; 5 GSK, Experimental Medicine Unit, Immunoinflammation TA, Medicines Research Centre, Stevenage, Herts SG1 2NY UK

**Keywords:** Soluble CTLA-4, Systemic lupus erythematosus, Immune regulation

## Abstract

**Background:**

The inhibitory CTLA-4 molecule is a crucial regulator of immune responses and a target for therapeutic intervention in both autoimmunity and cancer. In particular, CTLA-4 is important in controlling antigen-specific immunity, including responses to autoantigens associated with autoimmune disease. Here, we investigate cytokine responses to a range of lupus-associated autoantigens and assess whether the alternatively spliced isoform of CTLA-4, soluble CTLA-4 (sCTLA-4), contributes to immune regulation of autoantigen-specific immunity in systemic lupus erythematosus (SLE).

**Methods:**

The cell culture supernatant production of sCTLA-4 as well as the cytokines IL-10, IFN-γ, and IL-17 from peripheral blood mononuclear cells (PBMC) from lupus patients and age- and sex-matched healthy volunteer donors were measured in response to previously identified histone and small nuclear ribonucleoprotein (snRNP) autoantigen-derived peptides (H3_91-105_, H4_71-93_, and U170K_131-151_) by ELISA. We also examined the functional contribution of sCTLA-4 to immune regulation in the context of these autoantigenic peptides following blockade of sCTLA-4 with a selective anti-sCTLA-4 monoclonal antibody, JMW-3B3.

**Results:**

We identified responses to autoantigenic peptides, which revealed qualitative differences in cytokine (IL-10, IL-17, and IFN-γ) profiles between SLE patients and healthy donors. PBMC from healthy donors responded to each of the lupus peptides by secreting IFN-γ and IL-17, but PBMC from SLE patients produced IL-10. Although we did not observe differences in the levels of serum or PBMC culture supernatant sCTLA-4 in either cohort, blockade of sCTLA-4 in PBMC cultures responding to antigen enhanced the cytokine profiles associated with each group.

**Conclusion:**

The results show that lupus autoantigen-derived peptides display varied immunogenicity in lupus versus healthy volunteer donors, while sCTLA-4 acts to regulate the T-cell activity independently of response profile.

**Electronic supplementary material:**

The online version of this article (doi:10.1186/s13075-016-1075-1) contains supplementary material, which is available to authorized users.

## Background

Systemic lupus erythematosus (SLE) is a multisystem autoimmune disorder characterised by a range of clinical manifestations including arthritis, nephritis, central nervous system disease, alopecia, and photosensitivity. The disease is most prominent among women in their child-bearing years and, despite recent improvements in therapeutic options, many patients experience poor treatment outcomes [1]. At a molecular level, dysregulated apoptosis, inefficient clearance of cellular debris, and enhanced cytokine activity, notably interleukin (IL)-10 and interferon (IFN)-α, all appear to be important factors contributing to the inflammatory disease process [2].

Chronically activated autoantigen-specific T cells that promote production of high-affinity autoantibodies specific for nucleic acids/nucleic acid binding proteins are also factors in SLE [3–6]. These autoreactive T cells are driven primarily by recognition of self-peptides derived primarily from the same nucleic acid binding proteins that represent the main targets of the autoimmune response in lupus, e.g. histones [7]. Although these peptide autoantigens can drive pathological processes in lupus, interestingly they can also induce tolerance by influencing increased regulatory T-cell (Treg) activity, or by stimulating production of anti-inflammatory cytokines such as IL-10. The administration of self-peptides to promote tolerogenic responses in lupus has already been successful in murine models of lupus with one such peptide, Lupozor™, progressing through to clinical trials as a specific immunotherapy for the treatment of lupus [8, 9]. Peptides as therapeutics are thus very interesting because they are relatively cheap to produce and purify compared with biologics and can be more easily administered [10].

Analysis of cytokine responses in lupus patients driven by autoantigenic peptides has demonstrated a certain amount of heterogeneity in terms of the cytokine profiles stimulated by particular peptides, suggesting that some peptides may preferentially drive regulatory immune responses, e.g. by producing IL-10, and others a more typical effector T-cell cytokine response, e.g. IFN-γ [3], but while these response differences were identified in individual lupus patients it is unclear as to whether any particular profile is solely typical of lupus disease or if healthy donors also raise similar response patterns or any response at all to these lupus-associated peptides. Finally, the recent emergence of T helper (Th)17 T cells and their relevance to SLE raises the question of whether these peptides might drive IL-17 production [11].

Cytotoxic T lymphocyte antigen 4 (CTLA-4) is an inhibitory cell-surface receptor central to immune regulation and is predominantly expressed by Treg and activated CD4^+^ T-effector cells [12, 13]. CTLA-4 has become one of the most clinically exploited immune molecules of the last decade. Notably, augmenting CTLA-4 function by infusing the recombinant soluble form of CTLA-4 called CTLA4-Ig (or abatacept) selectively inhibits T-cell co-stimulation and is beneficial for treating some autoimmune diseases, notably rheumatoid arthritis [14]. CTLA4-Ig is currently approved for the treatment of rheumatoid arthritis, but in SLE its benefits have been more difficult to establish, even though its immunosuppressive effects were first clearly established in lupus mouse models [15]. A recent phase II clinical trial showed no overall patient benefit for this therapy, despite anecdotal reports that it benefited individual patients [16].

While natural CTLA-4 is generally considered as a cell-surface receptor, it can also be produced as a naturally secreted soluble molecule [17–19]. This natural soluble CTLA-4 (sCTLA-4) lacks the transmembrane domain of the full-length CTLA-4 receptor and carries a different C-terminal amino acid sequence, but crucially retains the capacity to engage with B7 ligands [18]. Thus, this secretable isoform has the potential to influence immune responses including those associated with autoimmune disease. Little is known of any regulatory role for natural sCTLA-4 in this respect although serum levels of sCTLA-4 are higher in SLE patient cohorts and other autoimmune diseases compared with healthy individuals [20, 21]. Furthermore, it is not known if sCTLA-4 might regulate autoantigenic T cells stimulated by self-peptides in lupus.

Here, we investigated the role of previously identified peptide autoantigens (H2B_10-33_, H3_91-105_, H4_71-93_, SmB_136-153_, and U170K_131-151_) [3, 22, 23] by comparing peripheral blood mononuclear cell (PBMC) responses derived from lupus patients or healthy volunteer study participants to determine whether particular effector or regulatory peptide driven responses are distinctive to lupus disease and, further, whether natural sCTLA-4 has a regulatory influence over those responses.

## Methods

### Donors and sample preparation

Blood samples were collected by venepuncture into heparan sulphate-containing vacutainers from lupus patients attending either the Rheumatology or Vasculitis department at Aberdeen Royal Infirmary (see Table 1 for demographic summary), and age- and sex-matched healthy volunteer donors with informed consent. Only patients with an existing diagnosis of SLE were recruited into the study. In addition, disease duration, evidence of existing disorders, serological analysis, and current medication were recorded. Age groups were clustered into four age ranges: 18–25, 26–35, 36–50, and 51+. Patients under the age of 18 or with pre-determined HIV status were excluded from the study. Ethical approval was obtained from the East of Scotland Research Ethics Committee (ref: REC 10/S1401/20). PBMC were prepared by Lymphoprep 1.077 (Axis Shield, Dundee, UK) density gradient centrifugation and cultured essentially as previously described [24] in RPMI 1640 medium (Invitrogen, Paisley, UK) supplemented with 5 % autologous human serum in an atmosphere of 37 °C, 5 % CO_2_; 1 × 10^6^ PBMC were cultured for 5 days in 1-ml wells unless otherwise stated.

**Table 1:** Demographic and clinical characteristics of the lupus patient study group

Demographic and clinical characteristics	Number of SLE patients
Age, years	48.1 ± 14.7
Sex, female/male	113/14
Renal disease	37 (29)
Arthritis	54 (43)
Serositis	38 (30)
Haematological disorder	82 (65)
Neurological disorder	24 (19)
Immunological disorder	119 (94)
Anti-dsDNA	40 (38)
ANA	104 (82)
Anti-phospholipid syndrome	18 (14)
Low C3	27 (22)
Low C4	44 (36)
Prednisolone <7.5 mg daily	39 (31)
Prednisolone >7.5 mg daily	17 (13)
Anti-malarials	82 (65)
Azathioprine, MTX, or mycophenolate mofetil	46 (36)
SLICC/ACR Damage Index, mean ± SD (range)	0.85 ± 1.2 (0–8)

Except where indicated otherwise, values are number (%) of patients

*Anti-dsDNA* anti-double-stranded DNA, *ANA* anti-nuclear antigen, *MTX* methotrexate, *SLE* systemic lupus erythematosus, *SLICC/ACR* Systemic Lupus International Collaborating Clinics/American College of Rheumatology

### T-cell stimuli and autoantigens

Histone peptides H2B (_10_PKKGSKKAVTKAQKKDGKKRKRSR_33_), H3 (_91_QSSAVMALQEASEAY_105_), and H4 (_71_TYTEHAKRKTVTAMDVVYALKRQ_93_), Spliceosomal peptide (SmB _136_GPSQQVMTPQGRGTVAAA_153_), and U1 small nuclear ribonucleoprotein of 70kDa (U1 70K _131_RIHMVYSKRSGKPRGYAFIEY_151_) were used in the non-phosphorylated form (GL Biochem, Shanghai, China). Peptides were at least 90 % pure as determined by reverse phase HPLC and Maldi-T of mass spectroscopy and dissolved in 5 % DMSO/endotoxin-free Hank’s balanced saline solution with phenol red (HBSS). Each peptide was each added to cell cultures at 5, 10, and 20 μg/ml, unless stated otherwise. Additionally, cells were stimulated with anti-CD3 mAb (OKT-3, 2 μg/ml), tuberculin purified protein derivative (PPD; Statens Serum Institut, Copenhagen, Denmark; 5 μg/ml), and Staphylococcal enterotoxin B (SEB; Sigma-Aldrich, Poole, Dorset, UK; 0.5 μg/ml).

### T-cell proliferative responses and antibody blockade

Cell proliferation was measured by ^3^H thymidine incorporation in duplicate samples using a 1450 Microbeta liquid scintillation counter (LKB Wallac, Turku, Finland). Results are presented as mean counts per minute (CPM).

Anti-sCTLA-4 blockade responses were performed with the JMW-3B3 anti-sCTLA-4 mAb, developed in-house at the University of Aberdeen. JMW-3B3 is an IgG1λ antibody that selectively binds sCTLA-4 and has previously been demonstrated to enhance antigen-specific immune responses compared with an IgG1 isotype control [25]. Enhanced responses were similar to initial adsorption experiments with AS-33P anti-CTLA-4 mAb (Antibody Solutions, Sunnyvale, CA, USA). Fresh antibody preparations were tested for the presence of endotoxin and stored without preservative at –20 °C prior to use.

### Cytokine ELISA

ELISA for cytokines in cell cultures was based on previously published methods [26]. Antibody pairs were: anti-IFN-γ (clones NIB42 and 4S.B3; BD Biosciences, Oxford, UK), anti-IL-17A (clones eBio64CAP17 and eBio64DEC17; eBiosciences, Hatfield, UK), anti-IL-10 (clones JES3-19 F1 and JES3-12G8; BD Biosciences, Oxford, UK), and IFN-α (clones MT1/3/5 and MT2/4/6; Mabtech, Sweden). Cytokine standards (IL-10, IL-17, IFN-γ) were from Peprotech EC Ltd. (London, UK), and IFN-α was from Mabtech. Bound antibody was detected using streptavidin-labelled alkaline phosphatase with a phosphate substrate (both Sigma Aldrich), and absorbance measured at 450 nm (corrected with a reference reading at 492 nm) with a Multiskan MS microplate photometer (Life and Laboratory Sciences, Basingstoke, UK). Cell culture supernatant levels of cytokine were measured following 5 days culture of PBMC at 37 °C, 5 % CO_2_.

### Soluble CTLA-4 ELISA

The selective ELISA for human sCTLA-4 used the anti-CTLA-4 murine mAb clone BNI3 (2 μg/ml) as a capture reagent, and biotinylated JMW-3B3 [25] as the sCTLA-4-specific detection reagent according to the protocol described for the cytokine ELISA above. Affinity-purified sCTLA-4 was used to construct standard curves.

### Data analysis

For greater clarity in the main figures, and for comparison of peptide- or control antigen-induced responses, all data are presented as Stimulation Indices (SI), i.e. the rates of stimulated versus resting cell responses. The full datasets are also presented in Additional file 1 together with a comprehensive statistical analysis (Prism GraphPad). SI values above 2 (i.e. double resting cell levels) were considered positive.

## Results

### Soluble CTLA-4 levels in serum and PBMC cell culture supernatants of lupus patients

Previous reports identified relatively high serum levels of sCTLA-4 in SLE patients [20, 21], but that work used antibodies capable of binding either native sCTLA-4, exocytosed full-length CTLA-4, or fragments of the extracellular domain of CTLA-4 cleaved from the cell surface. To ensure only sCTLA-4 and not cleaved products were detected, we used an antibody selective for a unique sequence of sCTLA-4 to analyse cell culture supernatant and serum levels of sCTLA-4 [25].

**Fig. 1:**
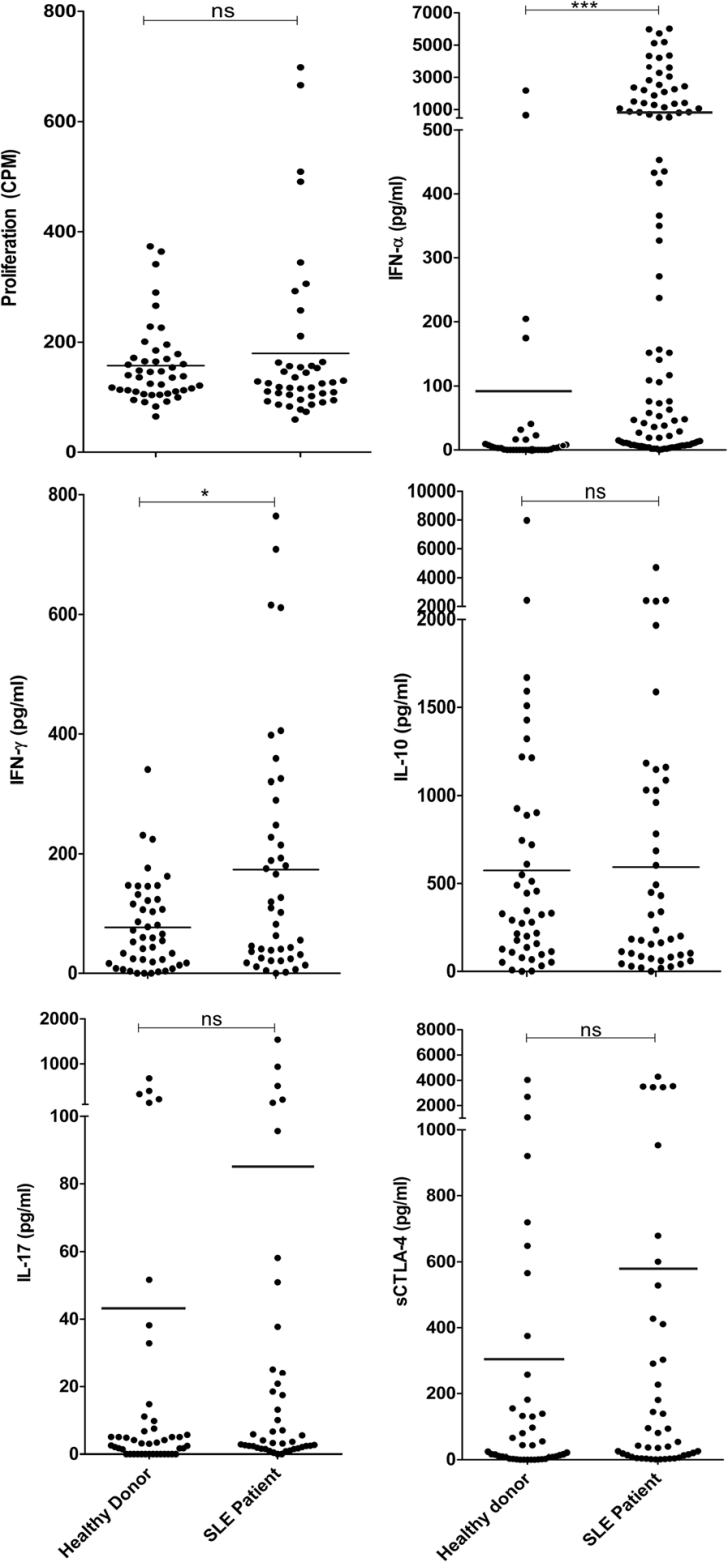
Resting levels of cellular proliferation and cell culture supernatant cytokines IFN-γ, IL-17, IL-10, IFN-α, and sCTLA-4 isolated from PBMC of SLE patients or age- and sex-matched healthy donors following incubation for 5 days (*n* = 45 per group; *p < 0.05, **p < 0.01, ***p < 0.001, non-parametric Mann-Whitney *U* test). *CPM* counts per minute, *IFN* interferon, *IL* interluekin, *ns* not significant, *sCTLA-4* soluble cytotoxic T lymphocyte antigen 4, *SLE* systemic lupus erythematosus

**Fig. 2:**
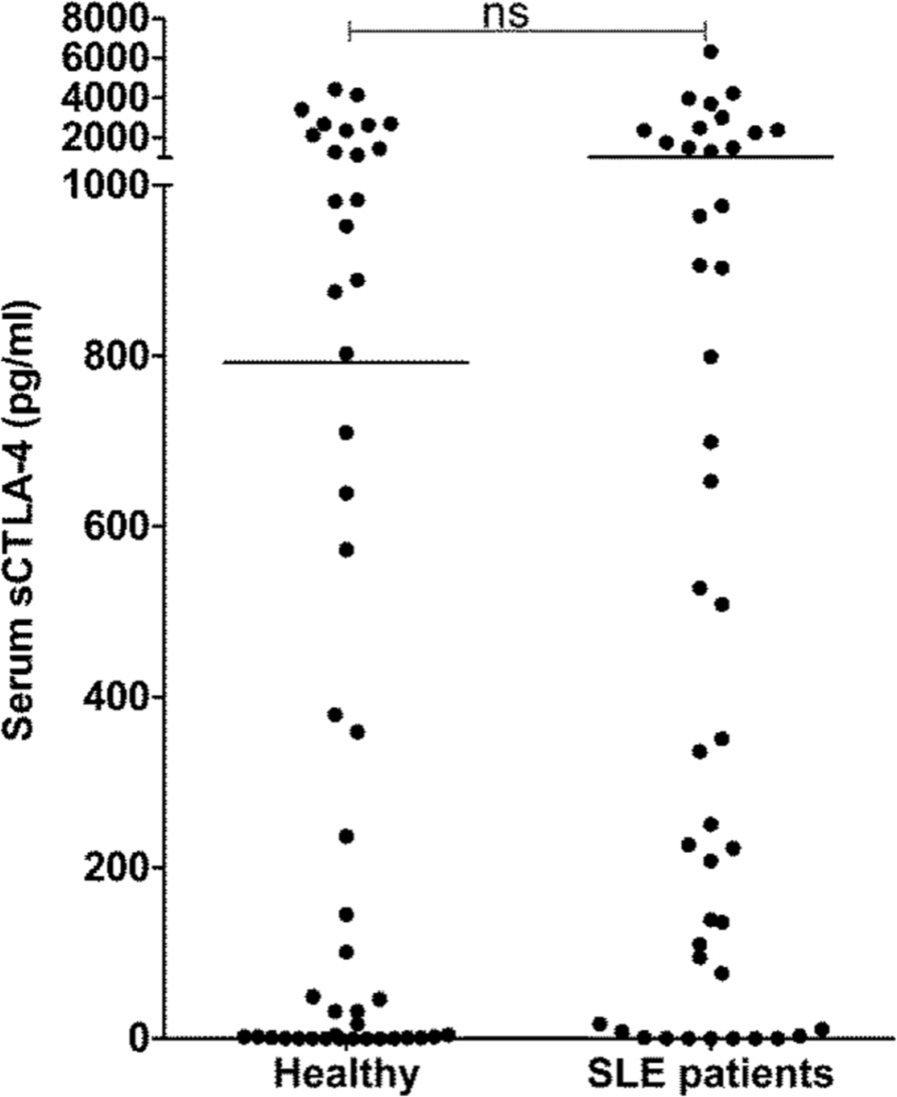
Serum levels of sCTLA-4 in SLE patients compared with age- and sex-matched healthy donors measured by ELISA (*n* = 45 per group; non-parametric Mann-Whitney *U* test). *ns* not significant, *sCTLA-4* soluble cytotoxic T lymphocyte antigen 4, *SLE* systemic lupus erythematosus

First, we measured levels of T helper-associated effector cytokines and sCTLA-4 in cell culture supernatants from resting PBMC, as well as sera from SLE patients and healthy donors and age- and sex-matched healthy volunteer donors (Fig. 1). As expected, median supernatant levels of IFN-γ and IFN-α in patient PBMC resting cell cultures were significantly higher than those of healthy volunteer donors (IFN-γ: 173.8 pg/ml versus 77.0 pg/ml, *p* < 0.05; IFN-α: 988.5 pg/ml versus 94.8 pg/ml, *p* < 0.0001; Mann-Whitney *U* test) but no significant difference in cellular proliferation (180 versus 158 CPM) or the cytokines IL-10 (668.9 pg/ml versus 702.3 pg/ml), IL-17 (87.7 versus 44.4 pg/ml), or sCTLA-4 (292.1 versus 552.5 pg/ml) was observed (Fig. 1). Levels of sCTLA-4 in supernatants from lupus patient PBMC varied widely between individuals (range 0–4288 pg/ml; Fig. 1). However, cell culture sCTLA-4 levels from the healthy donor cohort also varied widely (range 0–4027 pg/ml; Fig. 1). A similar pattern was observed in serum sCTLA-4 levels from SLE patients (range 0–6326 pg/ml, median 1044.0 pg/ml; Fig. 2) compared with sera from healthy donors (range 0–4421 pg/ml, median 792.4 pg/ml). In this analysis, in contrast to previous studies [20], despite an overall increase in serum levels of sCTLA-4, there was no significant difference between the healthy and patient donor cohort.

### Effect of sCTLA-4 on responses to peptide autoantigens in PBMC from lupus patients or healthy volunteer donors

Previously, sCTLA-4 was demonstrated to regulate cytokine responses in PBMC and T cells, but its regulatory effects in autoimmune disease have not been examined. Strong stimuli (e.g. anti-CD3 mAb) can temporarily suppress sCTLA-4 expression and production but, in healthy PBMC, more physiological stimuli (e.g. recall antigens) actually maintain sCTLA-4 levels and may even increase production [25]. To examine sCTLA-4 production in lupus, we investigated five peptide autoantigens identified in previous studies for their capacity to influence sCTLA-4 production.

**Fig. 3:**
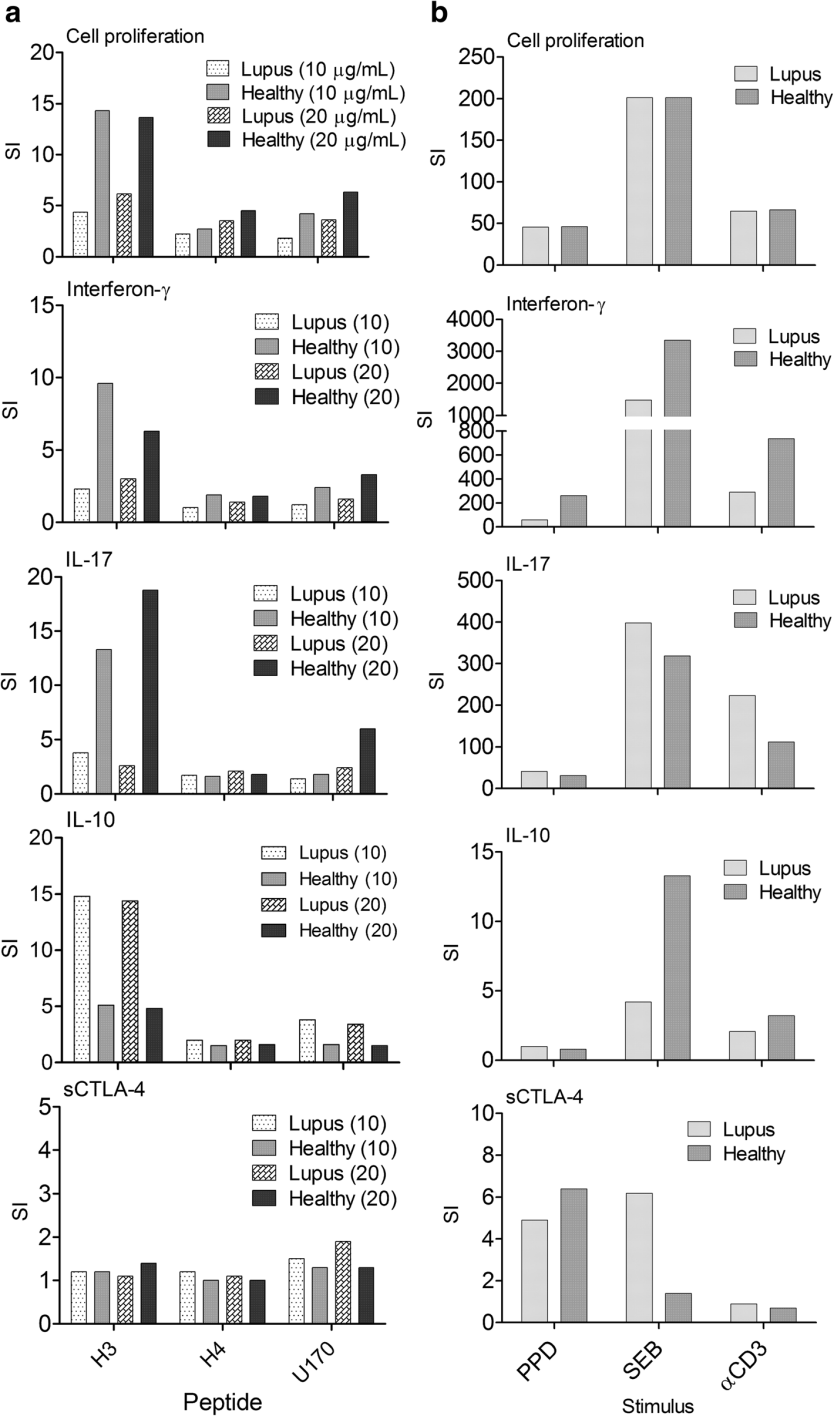
Analysis and summary of PBMC responses from healthy donors or SLE patients to peptide autoantigens H3_91-105_, H4_71-93_, and U170K_131-151_ at concentrations of 10 and 20 μg/ml (**a**) and PPD (5 μg/ml), SEB (0.5 μg/ml), and anti-CD3 mAb (2 μg/ml) control stimulants (**b**), before analysis of cellular proliferation, and levels of cell culture supernatant cytokine (IFN-γ, IL-17, IL-10, and sCTLA-4; *n* = 45; *p < 0.05 **p < 0.01, ***p < 0.001, non-parametric Mann-Whitney *U* test). Stimulation indices (*SI*), defined as fold-increase in response compared with resting cell controls, are shown for clarity with full data sets and statistical analysis shown in Additional file 1. *IL* interleukin, *ns* not significant, *PPD* purified protein derivative, *sCTLA-4* soluble cytotoxic T lymphocyte antigen 4, *SEB* Staphylococcal enterotoxin B, *CD* cluster of differentiation

Three of the five peptides examined (H3_91-105_, H4_71-93_, and U170K_131-151_; see Methods and materials for sequences) stimulated PBMC responses in the majority of both SLE patients and, unexpectedly, healthy donors, confirming their immunogenicity as determined by previous studies (summary of stimulation indices for H3_91-105_, H4_71-93_, and U170K_131-151_ are shown in Fig. 3a, and full individual datasets and statistical analysis are shown in Additional file 1, *n* = 45 per group). Two further peptides, H2B_10-33_ and SmB_136-153_, did not stimulate significant responses in either cohort.

Healthy donor PBMC proliferated vigorously and produced significantly higher amounts of the effector cytokines IFN-γ and IL-17 in response to peptides U170K_131-151_, H3_91-105_, and H4_71-93_ (*n* = 43; Fig. 3a and Additional file 1). Each of these peptides also induced lower but significant increases in IL-10 production compared with resting cells with a mean SI of 5 or less depending on the peptide and peptide dose. These healthy donors were, in effect, mounting a response characteristic of a mixed Th1/Th17 T-cell response to these lupus peptide autoantigens.

In contrast to healthy donors, SLE patient responses were characterised by increased production of IL-10 to the peptides rather than increased IFN-γ and IL-17. Despite production of immunosuppressive IL-10, PBMC from the SLE patient cohort significantly increased levels of cell proliferation to the peptides compared with resting cells, although levels were significantly lower than those observed in healthy donors (*p* < 0.01, Mann Whitney). Thus, this initial assessment of anti-peptide cytokine responses revealed a clear differentiation in cytokine profile between healthy and SLE patient donors.

We concurrently examined sCTLA-4 supernatant levels and, despite individual donors mounting large sCTLA-4 increases to the peptides, as a population both patient and healthy donor levels of sCTLA-4 were not significantly increased or decreased (Additional file 1).

To determine whether lupus patients would respond in the same way as healthy donors regarding sCTLA-4 production by PBMC stimulated by antigens rather than autoantigens, we examined a panel of stimulants, including PPD recall antigen, SEB superantigen, and agonist anti-CD3 mAb (Fig. 3b). Mean SI levels of sCTLA-4 were generally higher upon stimulation with PPD recall antigen in both patient and healthy donor cohorts and SEB in the lupus patient cohort, although levels were not statistically significant compared with resting PBMC (Wilcoxon). Further analysis revealed that the mean increase in SI primarily came from just a few individual donors that responded to PPD by secreting increased amounts of sCTLA-4 in an antigen-dependent manner. Reduction of sCTLA-4 by stimulation of PBMC with high doses of anti-CD3 mAb has been observed [17, 19] (Fig. 3b and Additional file 1) and was similar here [25].

### Effect of anti-sCTLA-4 antibody blockade on autoantigen-specific immune responses in lupus patients

Previously, we demonstrated in healthy donors that naturally secreted sCTLA-4 contributes to the regulation of antigen-specific effector T-cell responses by regulatory T cells, and that blocking its activity with a soluble CTLA-4 selective antibody can enhance T-effector response intensity compared with an IgG1 isotype control [25]. Little is known, however, whether amplification of such responses is intact in the context of autoimmunity. We therefore investigated the effects of sCTLA-4 blockade in lupus patients or healthy volunteer donors. Blockade of sCTLA-4 with the anti-sCTLA-4 mAb JMW-3B3 reduced supernatant levels of detectable sCTLA-4 by more than 92 % (*n* = 24 donors; *p* < 0.0001, Wilcoxon).

**Fig. 4:**
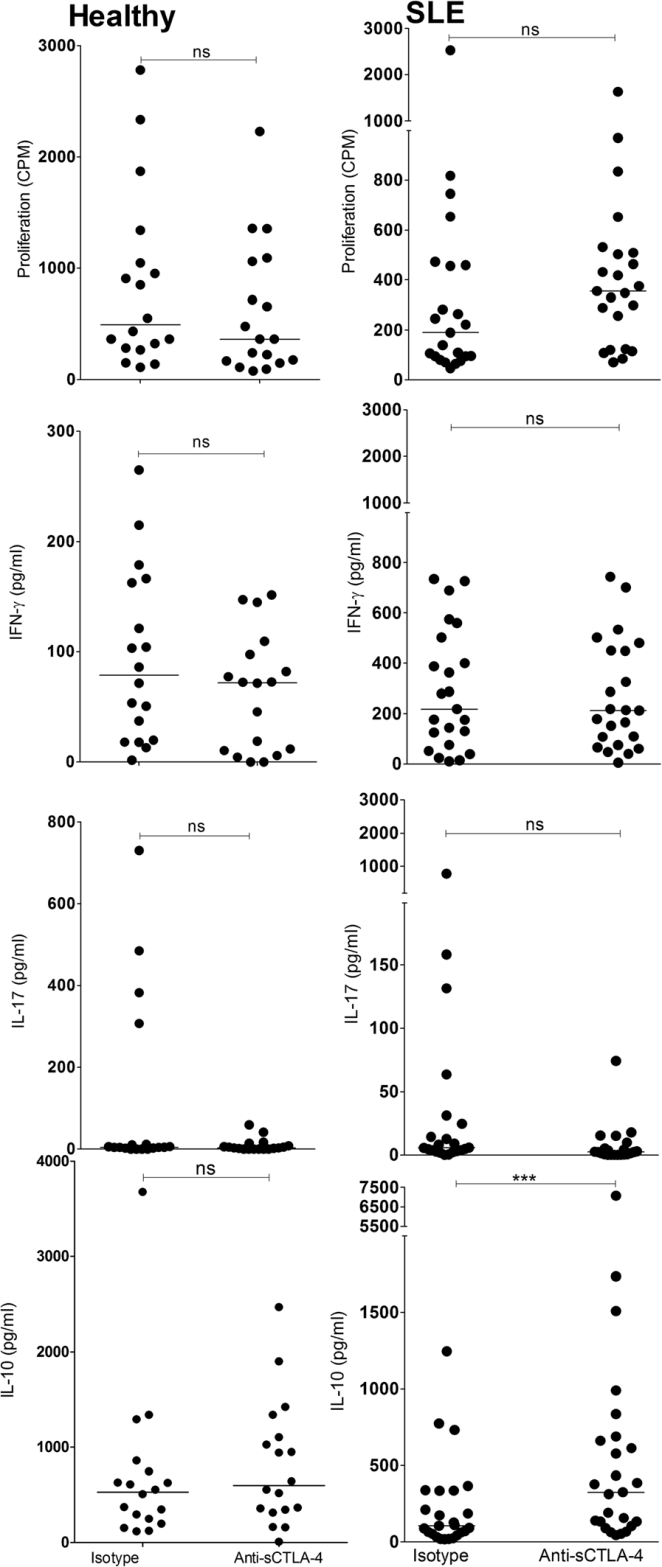
Effect of anti-sCTLA-4 mAb blockade on immune responses to H4_71-93_ peptide isolated from PBMC of healthy donors (*left panels*) or SLE patients (*right panels*). PBMC were incubated with H4_71-93_ (20 μg/ml) in the presence of JMW-3B3 anti-sCTLA-4 mAb or an IgG1 isotype antibody (both 10 μg/ml) for 5 days before analysis of cellular proliferation and levels of cell culture supernatant cytokines (IFN-γ, IL-17, IL-10, and sCTLA-4; *n* = 45 per group; *p < 0.05, **p < 0.01, ***p < 0.001, non-parametric Mann-Whitney *U* test; median values are shown). *CPM* counts per minute, *IFN* interferon, *IL* interluekin, *ns* not significant, *sCTLA-4* soluble cytotoxic T lymphocyte antigen 4, *SLE* systemic lupus erythematosus

We first assessed whether sCTLA-4 inhibition in cell cultures stimulated with lupus peptide autoantigens would affect any aspect of effector T-cell activity. Inhibition of sCTLA-4 in healthy donor cell cultures stimulated with U170K_131-151_, H3_91-105_, and H4_71-93_ had mixed effects on effector cytokine production and cell proliferation specific to each peptide, and were generally also individual to particular donors (summarised in Table 2 for both healthy and lupus donors). Blockade of sCTLA-4 in the healthy donor cohort generally suppressed or did not alter cell proliferation and cytokine production (Fig. 4 and summarised in Table 2; *p* < 0.05, Wilcoxon), while the SLE cohort following sCTLA-4 inhibition significantly enhanced the prevailing IL-10 cytokine phenotype stimulated by the peptides. Overall, our assessment of the effects of sCTLA-4 blockade on anti-peptide PBMC responses from healthy and lupus donors indicates that PBMC from the lupus cohort were more sensitive to changes in IL-10 levels following removal of the suppressive effects of sCTLA-4 suggesting that, nevertheless, sCTLA-4 suppresses effector T-cell responses independent of the profile of cytokine(s) it is producing.

**Table 2:** Summary of anti-sCTLA-4 mAb blockade effect on healthy donor and SLE patient peripheral blood mononuclear cell responses to control antigens and three stimulatory peptide autoantigens (H3_91-105_, H4_71-93_, and U170K_131-151_)

Peptide	Dose	Proliferation		IFN-γ		IL-17		IL-10	
	(μg/ml)	Healthy	SLE	Healthy	SLE	Healthy	SLE	Healthy	SLE
Resting^†^	-	ns	***⇩	ns	ns	ns	ns	ns	***⇧
U170K	10	*⇩	**⇩	ns	ns	ns	*⇩	ns	**⇧
	20	*⇩	*⇩	ns	ns	ns	**⇩	ns	ns
H3	10	ns	ns	ns	ns	ns	*⇩	ns	ns
	20	ns	**⇧	ns	ns	ns	ns	*⇩	*⇩
H4	10	**⇩	ns	ns	ns	*⇩	ns	*⇩	***⇧
	20	ns	ns	ns	ns	ns	ns	ns	***⇧
PPD	5	**⇧	ns	***⇧	ns	ns	ns	ns	*⇧
αCD3	5	ns	*⇩	ns	**⇩	ns	*⇩	ns	ns

*p ≤ 0.05, **p ≤ 0.01, ***p ≤ 0.001

*CD* cluster of differentiation, *H3*, histone 3, *H4* histone 4, *IFN* interferon, *IL* interleukin, *ns* not significant, *PPD* purified protein derivative, *SLE* systemic lupus erythematosus, *U170K* U1 small nuclear ribonucleoprotein 70 kDa

† = resting PBMC at day 5. Arrows next to probabilty asterix represent significant increase or decrease in test sample

**Fig. 5:**
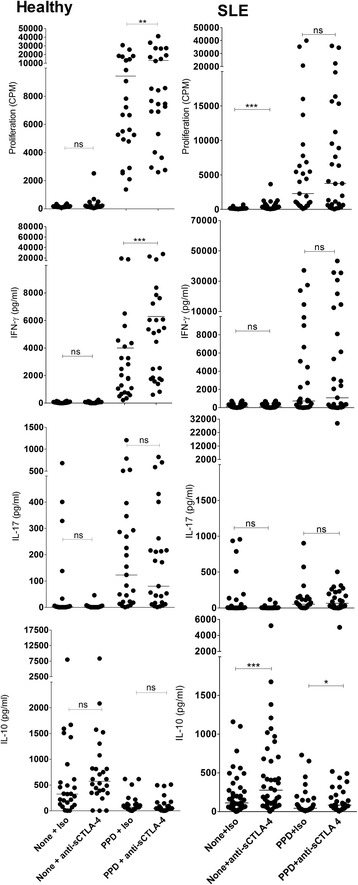
Effect of anti-sCTLA-4 mAb blockade on PBMC immune responses to PPD recall antigen. PBMC were incubated with the stimulants in the presence of JMW-3B3 anti-sCTLA-4 mAb or an IgG1 isotype antibody (both 10 μg/ml) for 5 days before analysis of cellular proliferation and levels of cell culture supernatant cytokines (IFN-γ, IL-17, IL-10, and sCTLA-4; *n* = 45 per group; *p < 0.05, **p < 0.01, ***p < 0.001, non-parametric Mann-Whitney *U* test; median values are shown). *CPM* counts per minute, *IFN* interferon, *IL* interluekin, *ns* not significant, *PPD* purified protein derivative, *sCTLA-4* soluble cytotoxic T lymphocyte antigen 4

An interesting corollary to the investigation of sCTLA-4 blockade on peptides was the same assessment of the effects on recall responses in lupus patients compared with healthy donors, where healthy donor PBMC were enhanced to PPD recall antigen following sCTLA-4 inhibition (Fig. 5 and Table 2), but there were no significant changes in levels of IL-17 or IL-10 following blockade of sCTLA-4. In contrast, sCTLA-4 blockade in resting PBMC from SLE patients raised both cell proliferation and IL-10 levels (Fig. 5 and Table 2), suggesting that PBMC in lupus patients seem primed to secrete higher levels of IL-10.

## Discussion

In this study we conducted a comprehensive review of PBMC responses to peptide autoantigens associated with SLE, and for the first time demonstrated qualitative differences in the responsive cytokine profile to autoantigenic peptides between SLE patients and healthy donors, together with an analysis of a role for sCTLA-4. In our study, of five autoantigenic peptides previously identified to contain T-cell stimulatory epitopes [3, 22, 23], three (H3_91-105_, H4_71-93_, and U170K_131-151_) were universally immunogenic for both SLE patients and healthy volunteer donors, allowing us to observe that lupus patients respond very differently to peptide autoantigens compared with healthy donors. This difference also extended to the immunoregulatory function of sCTLA-4, because removing its regulatory function accentuated differences between healthy and patient donor responses.

Soluble CTLA-4 is derived from an alternatively spliced isoform of full-length CTLA-4 and is secreted by several cell types including Treg [25], monocytes [27], melanoma cell lines [28], and pituitary gland cells [29], but its effects remain largely unexplored despite the clinical relevance of CTLA-4 both in autoimmune disease and cancer. High sCTLA-4 serum levels have previously been identified in lupus patients, and low efficiency of production was mooted to be associated with a susceptibility CT60 SNP genotype of CTLA-4 [30, 31].

The question here was could lupus-associated peptide autoantigens induce increased levels of sCTLA-4 in either patients or healthy donors? The answer was yes, but only in particular individuals. The question of why some individuals have the capacity to increase sCTLA-4 in response to antigens points to a particular primed T-cell subset with that capacity. Identifying methods for isolating and expanding such T-cell populations could offer a way forward for future understanding of immune regulation in SLE.

Blockade of sCTLA-4 revealed significant response differences between PBMC from healthy donors and SLE patients with only IL-10 levels increasing following PPD or peptide stimulation in the SLE patient group, suggesting that any response enhancement by blocking sCTLA-4 function is likely to reflect the predominant T-cell phenotype and the associated cytokine response, in this case IL-10. This points to a more general sCTLA-4 effect in which it may regulate the activity of antigen-specific effector responses but does not drive, in the short term at least, a skewing towards a particular cytokine profile produced by those activated T cells. In effect, sCTLA-4 most probably exerts its immunosuppressive effect by simply blocking B7/CD28 interactions, thus raising the threshold before which successful T-cell co-stimulation can occur.

Regarding the use of recombinant CTLA-4 products, e.g. CTLA4-Ig, in SLE, a key question is whether natural sCTLA-4 is functionally comparable. Previous studies identified sCTLA-4 to engage with B7 molecules and to effectively compete with CTLA4-Ig for binding [18, 32], but there are no hard data on relative binding affinities of sCTLA-4 with the full-length CTLA-4 receptor for B7 ligands. Our initial analysis of affinity-purified and recombinant sCTLA-4 structure indicates that it forms functional dimers mediated by a cysteine residue in the C-terminal amino acid sequence unique to sCTLA-4 (manuscript in preparation). This makes it more likely that affinity of sCTLA-4 for B7.1/B7.2 is comparable with that of both full-length CTLA-4 receptor and recombinant CTLA4-Ig. It is likely, therefore, that sCTLA-4 is functionally relevant to immune responses, but how serum levels in patients might impact on any CTLA-4-based therapy is unknown.

Perhaps the most surprising aspect of this study was the identification of a clear difference in the profile of cytokines produced in response to the peptide autoantigens between healthy or lupus patient PBMC. Healthy donors responded to the peptides by proliferating and producing higher levels of both IFN-γ and IL-17, whereas PBMC from lupus patients also proliferated but secreted IL-10. This supports the notion that, in lupus, T-cell responses are acting to support autoreactive B-cell responses and the production of high-affinity IgG autoantibodies [33–36]. While IL-10 is generally considered an anti-inflammatory cytokine, serum levels are often raised in lupus patients where it can act as a potent B-cell growth factor, driving autoantibody production, class switching, and plasmablast differentiation [33, 35, 37]. Thus, IL-10 may be important for suppressing tissue damage from activated pro-inflammatory macrophages and neutrophil responses to immune complex deposits, but can also contribute indirectly to the development of pathogenic immune complexes by raising high-affinity anti-nucleic acid and nucleoprotein autoantibody levels, a process remote from the inflammatory lesion [33]. It is of great interest, therefore, that responsive peptide autoantigen-specific T cells are a source of this IL-10.

This study provides evidence of a role for sCTLA-4 in immune regulation, yet clearly we need to understand more about the mechanistic processes which drive its production in response to antigen. One key issue that remains to be addressed is what is the primary stimulus of sCTLA-4 production? It is likely that production of sCTLA-4 in response to antigen relies both on antigen-specific T-cell responses but also the environmental milieu. We have recently observed that TGF-β2 is an important stimulatory factor of sCTLA-4 and it is now important to identify other stimulatory or suppressive factors that regulate the production of sCTLA-4.

## Conclusions

SLE patients present different cytokine response patterns to lupus-associated autoantigenic peptides compared to healthy donors, secreting significantly higher levels of IL-10. The natural immunosuppressant, sCTLA-4, supresses antigen-specific cytokine responses and antibody blockade of its activity enhances cytokine responses in healthy and lupus patient donors, independent of which cytokine is being secreted. Some individual responses indicated that peptides can induce increased production of sCTLA-4 but this effect was not significant at a population level for lupus peptide autoantigens.

Immunostimulatory peptide autoantigens including those investigated here may, therefore, be pivotal in driving the disease process, raising the prospect that targeting antigen-specific T cells to actually produce less IL-10, and allowing the immune response to progress to full resolution, could be a viable route to reduce immune complex-mediated pathology in lupus.

## Abbreviations

CD, cluster of differentiation; CPM, counts per minute; CTLA-4, cytotoxic T lymphocyte antigen 4; CTLA4-Ig, cytotoxic T lymphocyte antigen 4 immunoglobulin fusion protein; H2B, histone 2B; H3, histone 3; H4, histone 4; IFN, interferon; IgG, immunoglobulin G; IL, interleukin; mAb, monoclonal antibody; PBMC, peripheral blood mononuclear cells; PPD, purified protein derivative; RPMI 1640, Roswell Park Memorial Institute 1640 medium; sCTLA-4, soluble cytotoxic T lymphocyte antigen 4; SEB, Staphylococcal enterotoxin B; SI, Stimulation Index (Indices); SLE, systemic lupus erythematosus; SmB, Smith antigen B; SNP, single nucleotide polymorphism; snRNP, small nuclear ribonucleoprotein; Th, T helper; Treg, regulatory T cell; U170K, U1 small nuclear ribonucleoprotein 70kDa

## Additional file

Additional file 1: Full analysis of individual patient and donor peptide response datasets used to generate the summary data presented in Fig. 3. (DOCX 495 kb)

## References

[1] Rahman A, Isenberg DA (2008). Systemic lupus erythematosus. N Engl J Med.

[2] Liu Z, Davidson A (2012). Taming lupus—a new understanding of pathogenesis is leading to clinical advances. Nat Med.

[3] Lu L, Kaliyaperumal A, Boumpas DT, Datta SK (1999). Major peptide autoepitopes for nucleosome-specific T cells of human lupus. J Clin Invest.

[4] Monneaux F, Lozano JM, Patarroyo ME, Briand JP, Muller S (2003). T cell recognition and therapeutic effect of a phosphorylated synthetic peptide of the 70K snRNP protein administered in MR/lpr mice. Eur J Immunol.

[5] Wu HY, Ward FJ, Staines NA (2002). Histone peptide-induced nasal tolerance: suppression of murine lupus. J Immunol.

[6] Kang HK, Chiang MY, Liu M, Ecklund D, Datta SK (2011). The histone peptide H4 71-94 alone is more effective than a cocktail of peptide epitopes in controlling lupus: immunoregulatory mechanisms. J Clin Immunol.

[7] Rosen A, Casciola-Rosen L (2009). Autoantigens in systemic autoimmunity: critical partner in pathogenesis. J Intern Med.

[8] Zimmer R, Scherbarth HR, Rillo OL, Gomez-Reino JJ, Muller S. Lupuzor/P140 peptide in patients with systemic lupus erythematosus: a randomised, double-blind, placebo-controlled phase IIb clinical trial. Ann Rheum Dis. 2012. 10.1136/annrheumdis-2012-202460 PMC3812851 23172751

[9] Muller S, Monneaux F, Schall N, Rashkov RK, Oparanov BA, Wiesel P, Geiger JM, Zimmer R (2008). Spliceosomal peptide P140 for immunotherapy of systemic lupus erythematosus: results of an early phase II clinical trial. Arthritis Rheum.

[10] Briand JP, Schall N, Muller S (2014). Generation of self-peptides to treat systemic lupus erythematosus. Methods Mol Biol.

[11] Yang J, Chu Y, Yang X, Gao D, Zhu L, Yang X, Wan L, Li M (2009). Th17 and natural Treg cell population dynamics in systemic lupus erythematosus. Arthritis Rheum.

[12] Brunet JF, Denizot F, Luciani MF, Roux-Dosseto M, Suzan M, Mattei MG, Golstein P (1987). A new member of the immunoglobulin superfamily--CTLA-4. Nature.

[13] Walker LS, Sansom DM (2011). The emerging role of CTLA4 as a cell-extrinsic regulator of T cell responses. Nat Rev Immunol.

[14] Kremer JM, Westhovens R, Leon M, Di Giorgio E, Alten R, Steinfeld S, Russell A, Dougados M, Emery P, Nuamah IF, Williams GR, Becker JC, Hagerty DT, Moreland LW (2003). Treatment of rheumatoid arthritis by selective inhibition of T-cell activation with fusion protein CTLA4Ig. N Engl J Med.

[15] Finck BK, Linsley PS, Wofsy D (1994). Treatment of murine lupus with CTLA4Ig. Science.

[16] Wofsy D, Hillson JL, Diamond B (2012). Abatacept for lupus nephritis: alternative definitions of complete response support conflicting conclusions. Arthritis Rheum.

[17] Magistrelli G, Jeannin P, Herbault N (1999). Benoit De Coignac A, Gauchat JF, Bonnefoy JY, Delneste Y. A soluble form of CTLA-4 generated by alternative splicing is expressed by nonstimulated human T cells. Eur J Immunol.

[18] Oaks MK, Hallett KM, Penwell RT, Stauber EC, Warren SJ, Tector AJ (2000). A native soluble form of CTLA-4. Cell Immunol.

[19] Oaks MK, Hallett KM (2000). Cutting edge: a soluble form of CTLA-4 in patients with autoimmune thyroid disease. J Immunol.

[20] Liu MF, Wang CR, Chen PC, Fung LL (2003). Increased expression of soluble cytotoxic T-lymphocyte-associated antigen-4 molecule in patients with systemic lupus erythematosus. Scand J Immunol.

[21] Wong CK, Lit LC, Tam LS, Li EK, Lam CW (2005). Aberrant production of soluble costimulatory molecules CTLA-4, CD28, CD80 and CD86 in patients with systemic lupus erythematosus. Rheumatology (Oxford).

[22] Monneaux F, Briand JP, Muller S (2000). B and T cell immune response to small nuclear ribonucleoprotein particles in lupus mice: autoreactive CD4(+) T cells recognize a T cell epitope located within the RNP80 motif of the 70K protein. Eur J Immunol.

[23] Talken BL, Schafermeyer KR, Bailey CW, Lee DR, Hoffman RW (2001). T cell epitope mapping of the Smith antigen reveals that highly conserved Smith antigen motifs are the dominant target of T cell immunity in systemic lupus erythematosus. J Immunol.

[24] Hall AM, Ward FJ, Vickers MA, Stott LM, Urbaniak SJ, Barker RN (2002). Interleukin-10-mediated regulatory T-cell responses to epitopes on a human red blood cell autoantigen. Blood.

[25] Ward FJ, Dahal LN, Wijesekera SK, Abdul-Jawad SK, Kaewarpai T, Xu H, Vickers MA, Barker RN (2013). The soluble isoform of CTLA-4 as a regulator of T-cell responses. Eur J Immunol.

[26] Devereux G, Hall AM, Barker RN (2000). Measurement of T-helper cytokines secreted by cord blood mononuclear cells in response to allergens. J Immunol Methods.

[27] Laurent S, Carrega P, Saverino D, Piccioli P, Camoriano M, Morabito A, Dozin B, Fontana V, Simone R, Mortara L, Mingari MC, Ferlazzo G, Pistillo MP (2010). CTLA-4 is expressed by human monocyte-derived dendritic cells and regulates their functions. Hum Immunol.

[28] Laurent S, Queirolo P, Boero S, Salvi S, Piccioli P, Boccardo S, Minghelli S, Morabito A, Fontana V, Pietra G, Carrega P, Ferrari N, Tosetti F, Chang LJ, Mingari MC, Ferlazzo G, Poggi A, Pistillo MP (2013). The engagement of CTLA-4 on primary melanoma cell lines induces antibody-dependent cellular cytotoxicity and TNF-alpha production. J Transl Med.

[29] Iwama S, De Remigis A, Callahan MK, Slovin SF, Wolchok JD, Caturegli P (2014). Pituitary expression of CTLA-4 mediates hypophysitis secondary to administration of CTLA-4 blocking antibody. Sci Transl Med.

[30] Ueda H, Howson JM, Esposito L, Heward J, Snook H, Chamberlain G, Rainbow DB, Hunter KM, Smith AN, Di Genova G, Herr MH, Dahlman I, Payne F, Smyth D, Lowe C, Twells RC, Howlett S, Healy B, Nutland S, Rance HE, Everett V, Smink LJ, Lam AC, Cordell HJ, Walker NM, Bordin C, Hulme J, Motzo C, Cucca F, Hess JF, Metzker ML, Rogers J, Gregory S, Allahabadia A, Nithiyananthan R, Tuomilehto-Wolf E, Tuomilehto J, Bingley P, Gillespie KM, Undlien DE, Ronningen KS, Guja C, Ionescu-Tirgoviste C, Savage DA, Maxwell AP, Carson DJ, Patterson CC, Franklyn JA, Clayton DG, Peterson LB, Wicker LS, Todd JA (2003). Gough SC. Association of the T-cell regulatory gene CTLA4 with susceptibility to autoimmune disease. Nature.

[31] Purohit S, Podolsky R, Collins C, Zheng W, Schatz D, Muir A, Hopkins D, Huang YH, She JX (2005). Lack of correlation between the levels of soluble cytotoxic T-lymphocyte associated antigen-4 (CTLA-4) and the CT-60 genotypes. J Autoimmune Dis.

[32] Simone R, Pesce G, Antola P, Rumbullaku M, Bagnasco M, Bizzaro N, Saverino D (2014). The soluble form of CTLA-4 from serum of patients with autoimmune diseases regulates T-cell responses. Biomed Res Int.

[33] Rousset F, Garcia E, Defrance T, Peronne C, Vezzio N, Hsu DH, Kastelein R, Moore KW, Banchereau J (1992). Interleukin 10 is a potent growth and differentiation factor for activated human B lymphocytes. Proc Natl Acad Sci U S A.

[34] Llorente L, Richaud-Patin Y, Fior R, Alcocer-Varela J, Wijdenes J, Fourrier BM, Galanaud P, Emilie D (1994). In vivo production of interleukin-10 by non-T cells in rheumatoid arthritis, Sjogren’s syndrome, and systemic lupus erythematosus. A potential mechanism of B lymphocyte hyperactivity and autoimmunity. Arthritis Rheum.

[35] Llorente L, Zou W, Levy Y, Richaud-Patin Y, Wijdenes J, Alcocer-Varela J, Morel-Fourrier B, Brouet JC, Alarcon-Segovia D, Galanaud P, Emilie D (1995). Role of interleukin 10 in the B lymphocyte hyperactivity and autoantibody production of human systemic lupus erythematosus. J Exp Med.

[36] Yin Z, Bahtiyar G, Zhang N, Liu L, Zhu P, Robert ME, McNiff J, Madaio MP, Craft J (2002). IL-10 regulates murine lupus. J Immunol.

[37] Llorente L, Richaud-Patin Y, Garcia-Padilla C, Claret E, Jakez-Ocampo J, Cardiel MH, Alcocer-Varela J, Grangeot-Keros L, Alarcon-Segovia D, Wijdenes J, Galanaud P, Emilie D (2000). Clinical and biologic effects of anti-interleukin-10 monoclonal antibody administration in systemic lupus erythematosus. Arthritis Rheum.

