# Supplementary material for: Immunoregulatory soluble CTLA-4 modifies effector T-cell responses in systemic lupus erythematosus
Source: Arthritis Res Ther. 2016 Aug 4;18:180. doi: 10.1186/s13075-016-1075-1 (PMC4973056; doi:10.1186/s13075-016-1075-1)
Supplement: Additional file 1: — Full analysis of individual patient and donor peptide response datasets used to generate the summary data presented in Fig. 3. (DOCX 495 kb) [file 13075_2016_1075_MOESM1_ESM.docx]

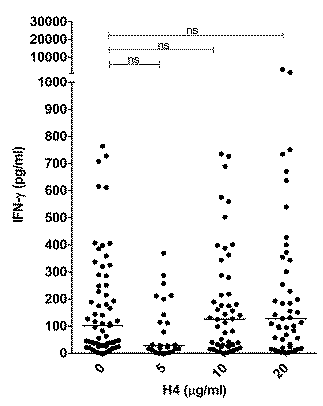

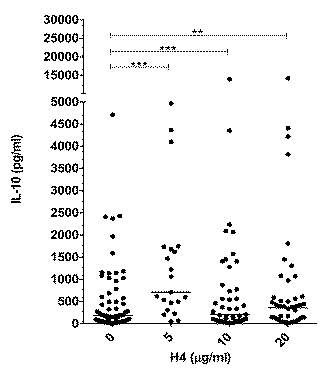

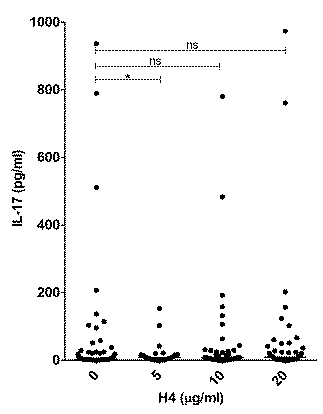

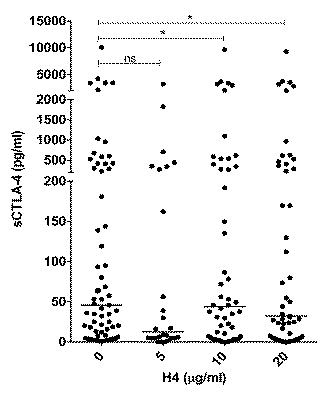

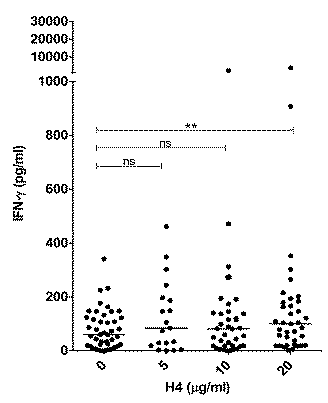

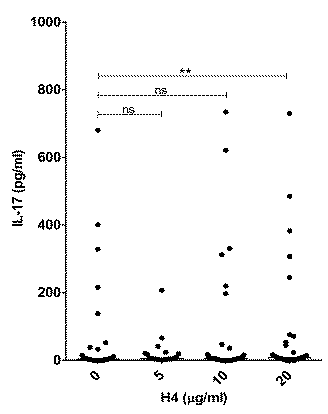

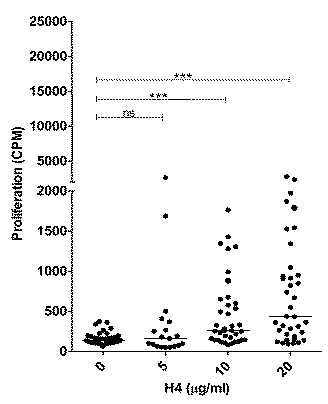

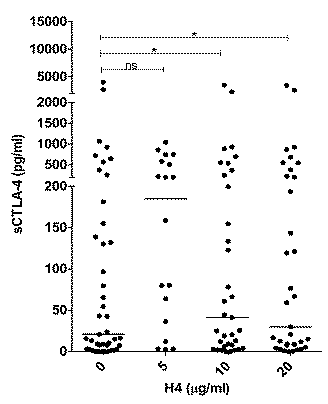

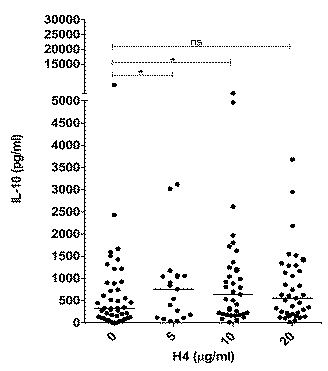

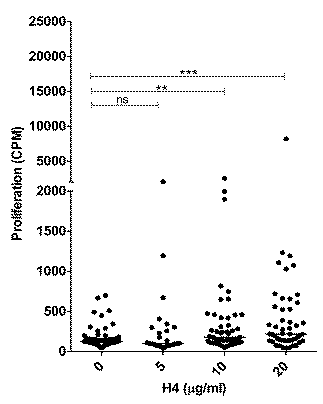


**Supplemental Figure 1**. Analysis of PBMC responses to the H4_71-93_ peptide derived from the U1 small nuclear ribonucleoprotein of 70kDa lupus autoantigen. PBMC were isolated from healthy donors (left panels) or SLE patients (right panels) and incubated for 5 days in the presence of H4_71-93_ peptide at concentrations of 5, 10 and 20 μg/ml before analysis of cellular proliferation and levels of cell culture supernatant cytokine (IFN-γ, IL-17, IL-10, IFN-α and sCTLA-4; n=45; * P<0.05 ** P<0.01, ***P<0.001, ns, not significant, non-parametric Mann-Whitney U test; median values are shown).

**Supplemental Figure 2**. Analysis of PBMC responses to the U170K_131-151_ peptide derived from the U1 small nuclear ribonucleoprotein of 70kDa lupus autoantigen. PBMC were isolated from healthy donors (left panels) or SLE patients (right panels) and incubated for 5 days in the presence of U170K_131-151_ peptide at concentrations of 5, 10 and 20 μg/ml before analysis of cellular proliferation and levels of cell culture supernatant cytokine (IFN-γ, IL-17, IL-10, IFN-α and sCTLA-4; n=45; * P<0.05 ** P<0.01, ***P<0.001, ns, not significant, non-parametric Mann-Whitney U test; median values are shown).


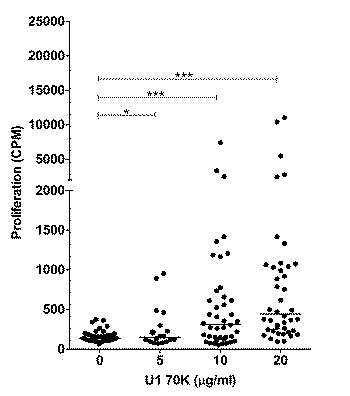

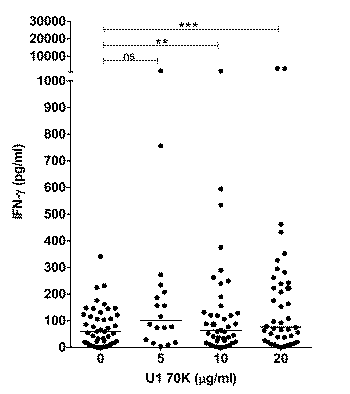

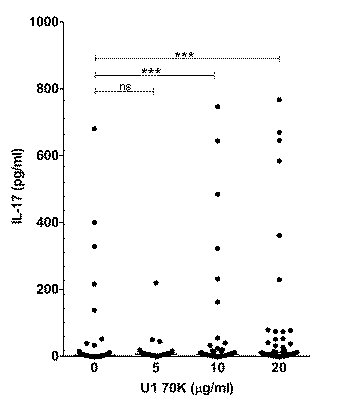

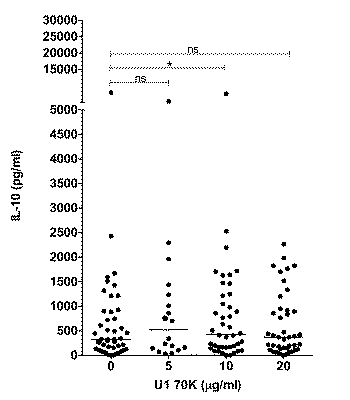

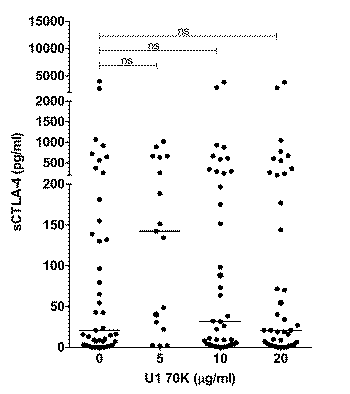

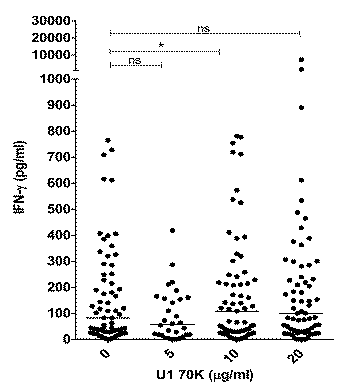

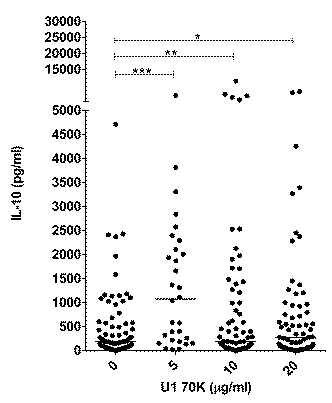

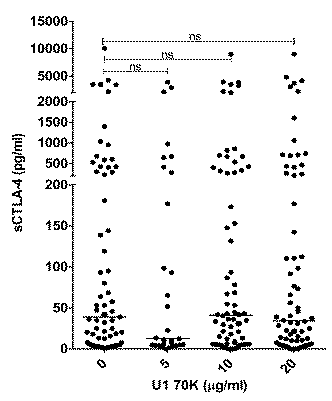

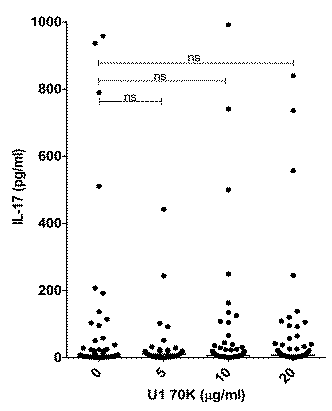

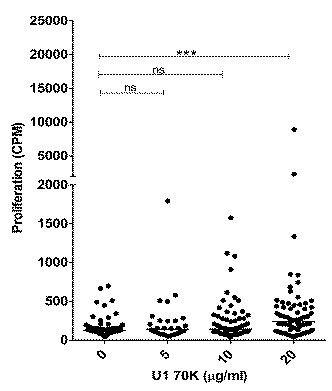


U1 70k_131-151_

**Supplemental Figure 3**. Analysis of PBMC responses to the H3_91-105_ peptide derived from the U1 small nuclear ribonucleoprotein of 70kDa lupus autoantigen. PBMC were isolated from healthy donors (left panels) or SLE patients (right panels) and incubated for 5 days in the presence of H3_91-105_ peptide at concentrations of 5, 10 and 20 μg/ml before analysis of cellular proliferation and levels of cell culture supernatant cytokine (IFN-γ, IL-17, IL-10, IFN-α and sCTLA-4; n=45; * P<0.05 ** P<0.01, ***P<0.001, ns, not significant, non-parametric Mann-Whitney U test; median values are shown).


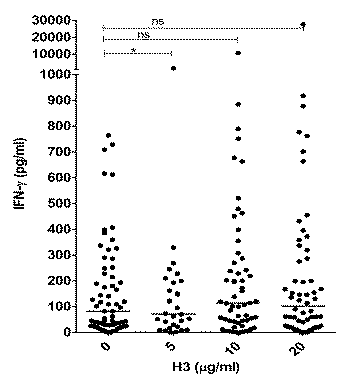

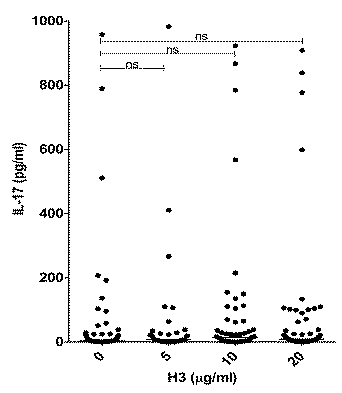

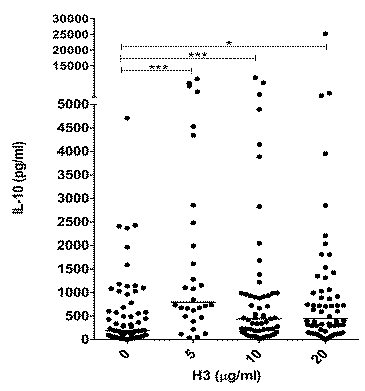

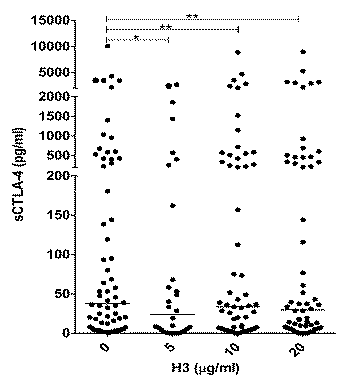

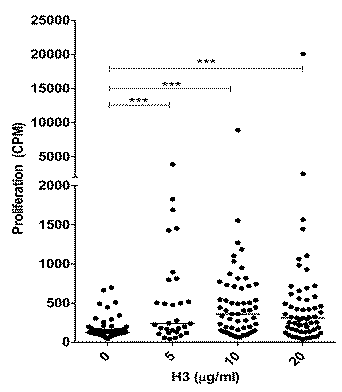

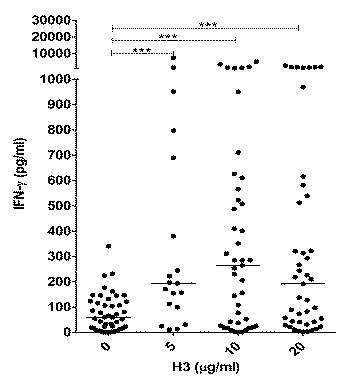

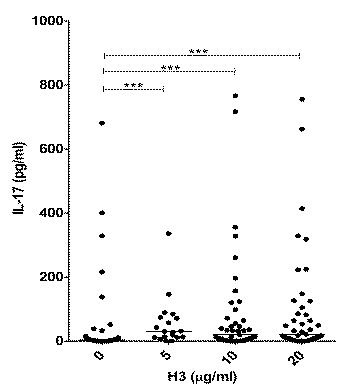

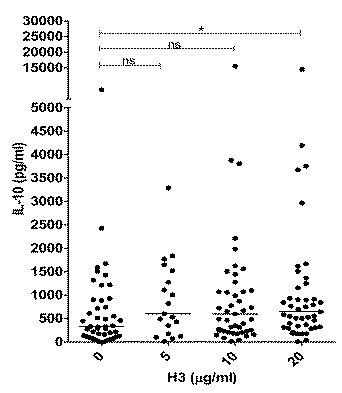

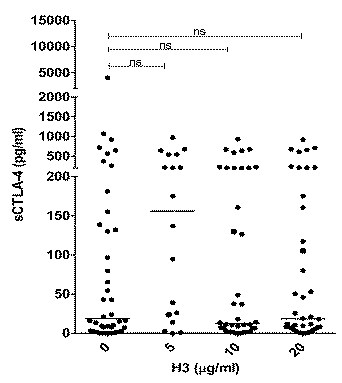

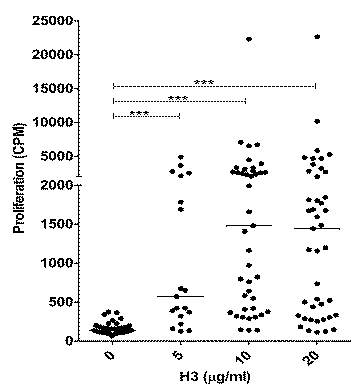


H3_91-105_

**Supplemental Figure 4** . Analysis of PBMC responses to a panel of control antigens and mitogens. PBMC were isolated from healthy donors (top panels) or SLE patients (lower panels) and incubated for 5 days before analysis of cellular proliferation and levels of cell culture supernatant cytokine (IFN-γ, IL-17, IL-10, IFN-α and sCTLA-4; n=45; * P<0.05 ** P<0.01, ***P<0.001, ns, not significant, P values determined by non-parametric Mann-Whitney U test; median values are shown).
